# Traumatic Pulmonary Arteriovenous Malformation Presenting as Spontaneous Hemothorax

**DOI:** 10.7759/cureus.16072

**Published:** 2021-06-30

**Authors:** Obteene Azimi-Ghomi, Marcela Ramirez, Dieter Brummund, Marc Gibber, Maurice R Mawad

**Affiliations:** 1 Department of General Surgery, Kendall Regional Medical Center, Miami, USA; 2 Department of Cardiothoracic Surgery, Aventura Hospital and Medical Center, Aventura, USA; 3 Department of Cardiothoracic Surgery, Kendall Regional Medical Center, Miami, USA

**Keywords:** pulmonary arteriovenous malformation, avm, pavm, hemothorax, thoracotomy

## Abstract

Pulmonary arteriovenous malformations (PAVM), also known as pulmonary arteriovenous fistulas, are abnormal connections between the pulmonary arterial and venous systems. The majority occur secondary to the congenital syndrome hereditary hemorrhagic telangiectasia (HHT), also known as Osler-Weber-Rendu disease. Trauma is an extremely rare etiology of PAVM, comprising less than 1% of all reported cases. Trauma can be associated with both immediate and delayed development of PAVM, and present similarly to PAVM associated with HHT. We report a case of a traumatic PAVM that developed in a patient one year following blunt thoracic trauma with a rib fracture. The patient subsequently developed a rupture of the PAVM, resulting in spontaneous hemothorax. She required multi-unit blood transfusion and multiple thoracostomy tube placements. The patient subsequently underwent a failed attempt at angioembolization of the PAVM. She eventually required a thoracotomy for surgical excision of the PAVM. We discuss the traumatic etiologies, clinical presentation, diagnostic assessments, and therapeutic modalities for the management of PAVM.

## Introduction

Pulmonary arteriovenous malformations (PAVM), also known as pulmonary arteriovenous fistulas, are abnormal connections between the pulmonary arterial and venous systems. The majority occur secondary to the congenital syndrome hereditary hemorrhagic telangiectasia (HHT), also known as Osler-Weber-Rendu disease [1-2]. Trauma is an extremely rare etiology of PAVM, comprising less than 1% of all reported cases [3-4]. Trauma can be associated with both immediate and delayed development of PAVM and present similarly to PAVM associated with HHT. We report a case of a traumatic PAVM that developed in a patient one year following blunt thoracic trauma with a rib fracture.

## Case presentation

A 76-year-old female with a past medical history of hypothyroidism and a fall one year prior, resulting in several right-sided rib fractures, presented to a free-standing emergency department with sudden onset chest pain and shortness of breath. The patient denied a history of a recent fall or trauma to the chest. Computed tomography (CT) of the chest, abdomen, and pelvis demonstrated a large right-sided hemothorax with a mediastinal shift. Chronic rib fractures of the right anterior third to fifth ribs were also noted.

**Figure 1:**
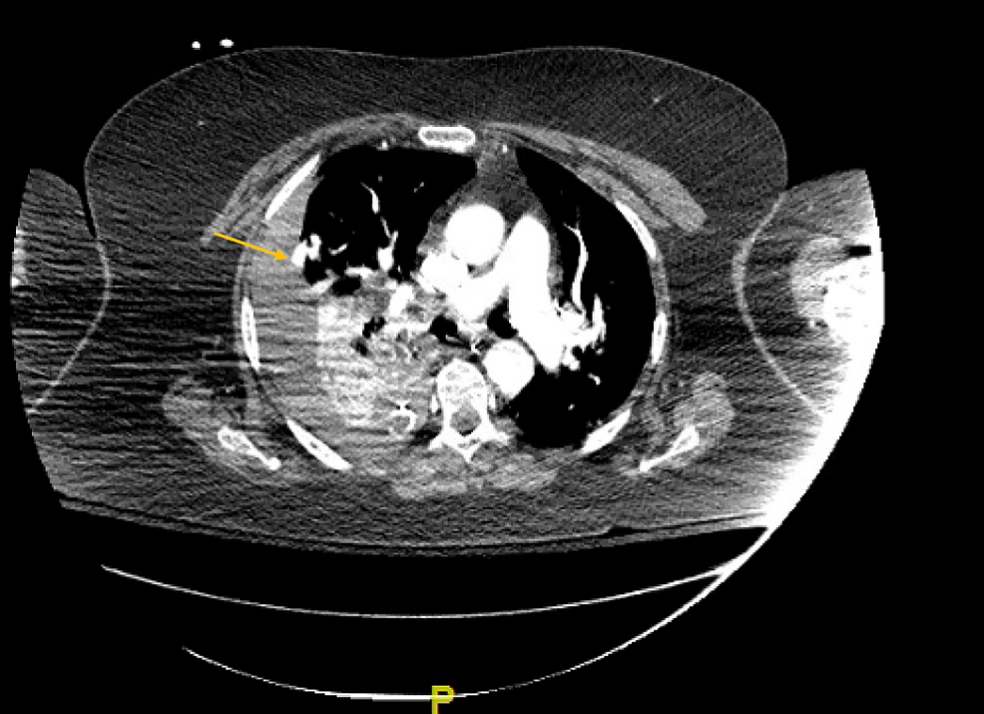
CT angiography demonstrating the arteriovenous malformation at the periphery of the right middle lobe (arrow). Large hemothorax can also be noted.

She underwent right-sided tube thoracostomy placement with the evacuation of several hundred milliliters (mL) of blood as she was transferred as a trauma alert to our facility. On arrival, she was tachycardic and hypotensive. Her hemoglobin was 11.2 mg/dL, and there was a lactic acidosis of 4.8 mmol/L. A right-sided chest tube was in place without further evacuation of blood noted. A portable chest X-ray demonstrated persistent large hemothorax with mediastinal shift, with the tube in a basilar position. A second tube thoracostomy was placed with immediate evacuation of 500 mL of blood. The patient was given two units of red blood cells, and her hemodynamics stabilized. She was taken for CT angiography (CTA) of the chest that demonstrated an arteriovenous malformation (AMV) at the periphery of the right middle lobe measuring 1.8 cm, located at the interface with the pleural (Figure 1). No active extravasation was noted. This AVM was noted to be immediately adjacent to the previous rib fractures. She was transferred to the trauma intensive care unit. Follow-up labs demonstrated stable hemoglobin and normalization of lactic acid levels.

**Figure 2:**
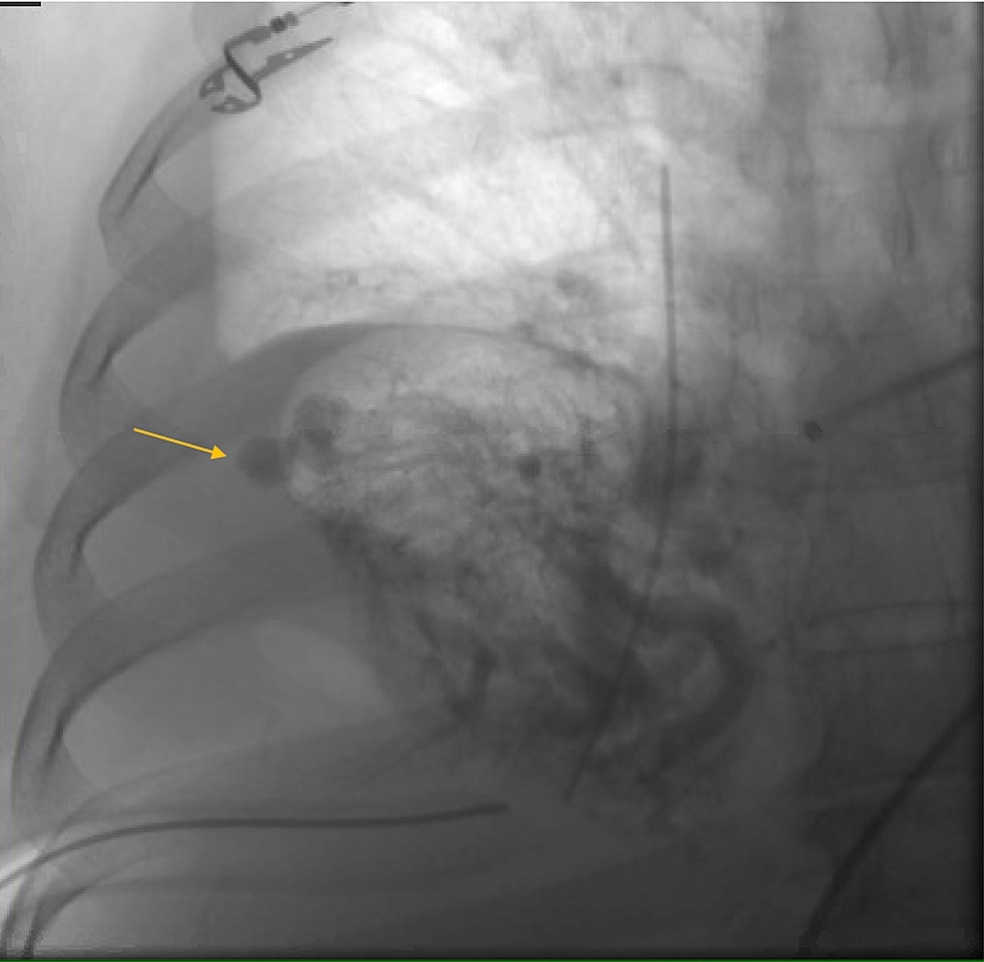
Angiogram of the right middle lobe demonstrating the arteriovenous malformation at the periphery of the lobe (arrow). The bilobed appearance and rapid venous filling can be noted.

Interventional radiology (IR) was consulted for embolization of the AVM. The patient underwent a right pulmonary catheterization the following day, and the AVM was confirmed with angiography, located at the peripheral aspect of the right middle lobe (Figure 2). IR was unable to catheterize and embolize the AVM due to feeding vessel tortuosity. A second attempt was made two days later by the IR team to angio-embolize the AVM, however, this was also unsuccessful.

**Figure 3:**
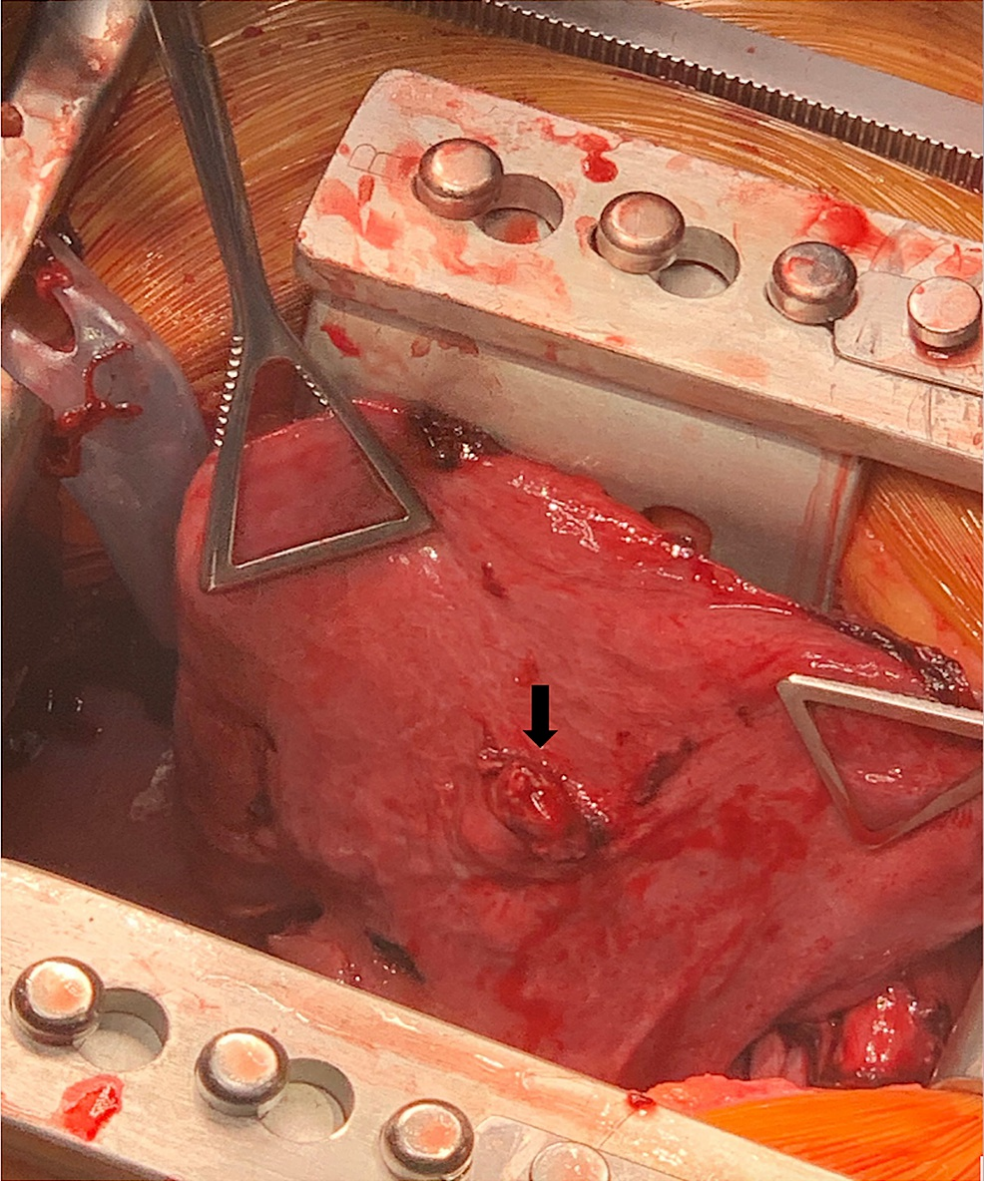
Intraoperative picture of the arteriovenous malformation in situ (arrow)

**Figure 4:**
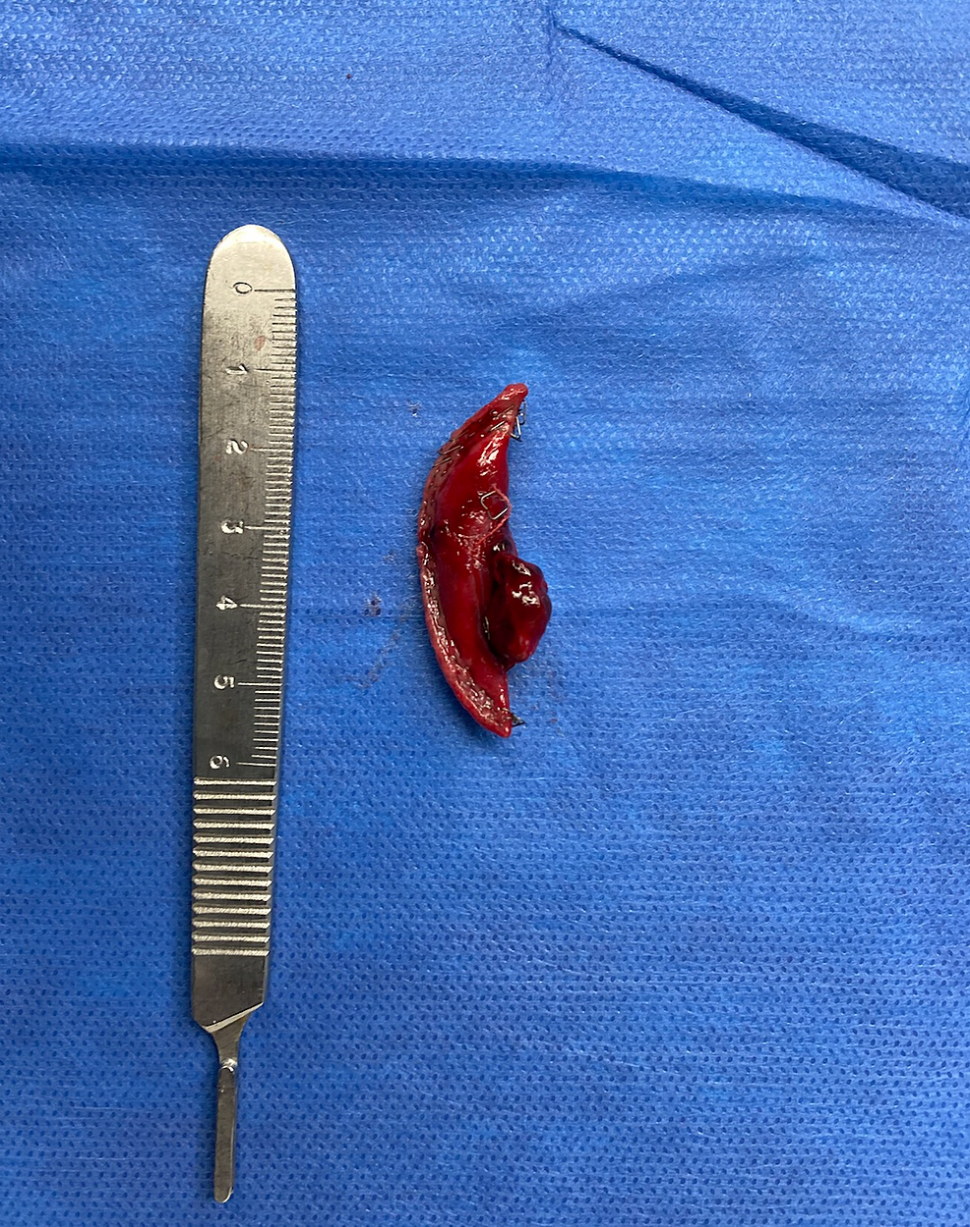
Resected arteriovenous malformation

Following a multidisciplinary discussion between the IR, Trauma, and Cardiothoracic surgery teams, a decision was made to proceed with open resection of the AVM. The patient subsequently underwent a right thoracotomy the following day, with the evacuation of the remaining hemothorax and clot within the chest. The bi-lobed AVM was noted at the periphery of the anterior aspect of the right middle lobe (Figure 3). There was no active bleeding from the AVM. A wedge resection of the involved lobe containing the AVM using a 60 mm endo-stapler was performed (Figure 4). The basilar chest tube was removed; the second chest tube was left in place. The patients’ postoperative hospital stay was uneventful. Her chest tube was removed on postoperative day #2 and she was discharged home on postoperative day #3.

## Discussion

Pulmonary arteriovenous malformations (PAVM), also known as pulmonary arteriovenous fistulas, are abnormal connections between the pulmonary arterial and venous systems [1-2]. The vast majority are congenital, with hereditary hemorrhagic telangiectasia (HHT) comprising 47%-88% of these cases [2]. Traumatic PAVM are a very rare form of PAVM. A retrospective review demonstrated that traumatic PAVM comprised <1% of all PAVM [3-4]. A literature review of traumatic PAVM demonstrated that 57% of cases reviewed occurred secondary to gunshot wounds [4-6]. Stabbings, rib fractures secondary to blunt thoracic trauma, and thoracic surgery have also been implicated as causes of traumatic PAVM [1-2,5-8]. Traumatic PAVM have been reported to present up to 30 years after the inciting trauma [7].

Approximately 13%-55% of patients with any type of PAVM are asymptomatic [1-2]. Patients with traumatic PAVM predominantly present with respiratory and cardiac symptoms, similar to those seen in congenital PAVM [1]. The most common presentations are dyspnea and refractory hypoxemia [1]. Hemoptysis, hemothorax, cyanosis, murmurs, or bruits over the site of the PAVM may also be seen [1-2]. PAVM located centrally tend to present with hemoptysis and signs of high-output heart failure, whereas peripherally located PAVM can present with pleuritic chest pain and hemothorax [1-3,5-6]. Neurological symptoms, commonly seen in congenital PAVM, are rare in the traumatic variant. Signs and symptoms specific to HHT, such as telangiectasias, epistaxis, and melena, are also absent.

Diagnosis of traumatic PAVM mirrors that of congenital PAVM. Plain chest X-rays demonstrate abnormalities in 98% of patients, whether demonstrating the PAVM or a resultant complication [2]. Contrast echocardiography with agitated saline has been described in the diagnostic workup of PAVM though it can only determine the presence/absence of PAVM and does not provide anatomical detail [1-2]. Contrast-enhanced CT is a valuable diagnostic tool in not only detecting the presence of PAVM but also in defining the anatomy [1,4]. CT angiography has been demonstrated to have higher sensitivity in the detection of PAVM compared to conventional angiography though the latter is better able to detail the vascular architecture of PAVM [1]. Pulmonary angiography remains the gold standard in the diagnosis of PAVM [1-2]. It is typically used after the initial diagnosis of PAVM, as it can confirm the diagnosis and define the arterial and venous architecture [1-2]. Depending on the location and structure, angiography can also be used therapeutically to embolize the PAVM [2,6,9].

Treatment of PAVM depends on symptomatology, location, size, and architecture. Treatment is typically recommended in all symptomatic patients and any asymptomatic patient with a PAVM <2 cm with feeder artery >3 mm in diameter [1-2]. Treatment is aimed at correcting any associated hypoxemia and high-output heart failure, as well as preventing the development or recurrence of hemorrhagic complications [4-6].

Surgical resection of the involved portion of the lung has historically been the standard of care, however, angiographic embolization has now become the preferred treatment modality, as it preserves otherwise healthy lung parenchyma [1,6]. The current preferred treatment is percutaneous angio-embolic therapy with coils and/or balloons [6,9]. These therapies function by targeting and occluding the feeder artery, thereby eliminating flow through the PAVM [1-2,9]. A retrospective review of 808 PAVM across 288 patients demonstrated a success rate of >99% (803/808) [9]. Coils were used more often than balloons though no studies demonstrate a difference in efficacy [9]. The most common complication of angioembolization is pleuritic chest pain, seen in 13%-31% of patients, and is usually self-limiting [9].

Surgical resection is indicated for patients who fail embolic therapy, have intrapleural rupture of PAVM, develop serious bleeding despite embolization, have PAVM not amenable to embolic therapy, or have serious contrast allergy [2,6-9]. Lobectomy was classically performed, however, local wedge resection or segmentectomy are now preferred, as they minimize resection of healthy lung tissue [8,10-12]. Video-assisted thoracoscopic surgery (VATS) is associated with decreased morbidity compared to open thoracotomy [10]. Surgical resection may be preferred to embolization in peripheral lesions, such as ours, which are inaccessible to embolic therapy, as well as lesions with associated hemothorax, as it also allows for the evacuation of pleural fluid [1,6,10-12]. Surgical therapy carries minimal morbidity and mortality when performed by trained and experienced surgeons. A review of five case series from 1969 to the present that contained a combined cohort of 99 patients demonstrated a 0% mortality rate [11-15].

## Conclusions

Traumatic PAVM are a rare form of PAVM that can develop following trauma to the chest and lungs. Blunt trauma resulting in rib fractures and lung laceration, as seen with outpatients, as well as penetrating trauma in the form of stabbing, gunshot wounds, and surgical procedures, are known to cause traumatic PAVM. These PAVM can present in both an immediate or delayed fashion with dyspnea, hypoxemia, heart failure, hemoptysis, and hemothorax. A thorough medical history documenting a history of trauma to the chest, an absence of HHT familial history, in conjunction with appropriate imaging, is essential in determining the diagnosis. Treatment depends on the location of the PAVM, with most located within the lung parenchyma and thus amenable to angiography and embolization. PAVM that are inaccessible to angioembolization develop bleeding despite embolization, and patients with refractory hemodynamic shock should undergo surgical exploration and resection of the PAVM. Recent case series demonstrate that with prompt surgical management, mortality rates approach 0%.
